# COVID-19: A Global Challenge with Old History, Epidemiology and Progress So Far

**DOI:** 10.3390/molecules26010039

**Published:** 2020-12-23

**Authors:** Mujeeb Khan, Syed F. Adil, Hamad Z. Alkhathlan, Muhammad N. Tahir, Sadia Saif, Merajuddin Khan, Shams T. Khan

**Affiliations:** 1 Department of Chemistry, College of Science, King Saud University, P.O. Box 2455, Riyadh 11451, Saudi Arabia; kmujeeb@ksu.edu.sa (M.K.); sfadil@ksu.edu.sa (S.F.A.); khathlan@ksu.edu.sa (H.Z.A.); 2 Department of Chemistry, King Fahd University of Petroleum and Minerals, P.O. Box 5048, Dhahran 31261, Saudi Arabia; muhammad.tahir@kfupm.edu.sa; 3 Department of Environmental Sciences, Kinnaird College for Women, Lahore 54000, Pakistan; sadia.saifpk@gmail.com; 4 Department of Agricultural Microbiology, Faculty of Agricultural Sciences, Aligarh Muslim University, Aligarh 202002, UP, India

**Keywords:** COVID-19, MERS-CoV, coronavirus

## Abstract

Humans have witnessed three deadly pandemics so far in the twenty-first century which are associated with novel coronaviruses: SARS, Middle East respiratory syndrome (MERS), and COVID-19. All of these viruses, which are responsible for causing acute respiratory tract infections (ARTIs), are highly contagious in nature and/or have caused high mortalities. The recently emerged COVID-19 disease is a highly transmittable viral infection caused by another zoonotic novel coronavirus named severe acute respiratory syndrome coronavirus 2 (SARS-CoV-2). Similar to the other two coronaviruses such as SARS-CoV-1 and MERS-CoV, SARS-CoV-2 is also likely to have originated from bats, which have been serving as established reservoirs for various pathogenic coronaviruses. Although, it is still unknown how SARS-CoV-2 is transmitted from bats to humans, the rapid human-to-human transmission has been confirmed widely. The disease first appeared in Wuhan, China, in December 2019 and quickly spread across the globe, infected 48,539,872 people, and caused 1,232,791 deaths in 215 countries, and the infection is still spreading at the time of manuscript preparation. So far, there is no definite line of treatment which has been approved or vaccine which is available. However, different types of potential vaccines and therapeutics have been evaluated and/or are under clinical trials against COVID-19. In this review, we summarize different types of acute respiratory diseases and briefly discuss earlier outbreaks of coronaviruses and compare their occurrence and pathogenicity with the current COVID-19 pandemic. Various epidemiological aspects of COVID-19 such as mode of spread, death rate, doubling time, etc., have been discussed in detail. Apart from this, different technical issues related to the COVID-19 pandemic including use of masks and other socio-economic problems associated with the pandemic have also been summarized. Additionally, we have reviewed various aspects of patient management strategies including mechanism of action, available diagnostic tools, etc., and also discussed different strategies for the development of effective vaccines and therapeutic combinations to deal with this viral outbreak. Overall, by the inclusion of various references, this review covers, in detail, the most important aspects of the COVID-19 pandemic.

## 1. Introduction

Recently, the outbreak of various infectious diseases has significantly impacted the lives of millions of people [1,2]. These diseases have not only strained our medical and public health facilities but also burdened economists, scientists, and politicians in responding to the financial hardships, discovery of vaccines, and dealing with public anxieties and expectations, respectively [3,4,5,6]. One such infectious disease occurred at the end of 2019, just before the biggest Chinese festival [7]. This was noticed by the sudden emergence of several acute pneumonia cases with similar symptoms in Wuhan, one of the largest cities in China [8,9]. Later, the cause of the disease was identified by genome sequencing technology as a novel form of coronavirus which was named as severe acute respiratory syndrome coronavirus 2 (SARS-CoV-2) and the disease was named as coronavirus disease 2019, or COVID-19 [10]. After the emergence of SARS-CoV (severe acute respiratory syndrome coronavirus) and Middle East respiratory syndrome coronavirus (MERS-CoV), SARS-CoV-2 is another member of coronavirus family that has a strong ability to infect human beings [11,12]. As of 5 November 2020, a total of 48,539,872 infected cases with 1,232,791 confirmed deaths have been reported in 215 countries and territories around the world resulting from COVID-19 [13]. This disease has caused a massive global health challenge and has created ripples in the medical fraternity [14]. Undoubtedly, it requires unprecedented strategies such as massive surveillance to prevent spreading, creation of a sophisticated network of diagnostics and medical facilities for immediate detection and treatment of the disease, and extensive research for the quick development of drugs and vaccines for future protections [15,16]. 

## 2. History of Respiratory Viruses Outbreaks

**Figure 1:**
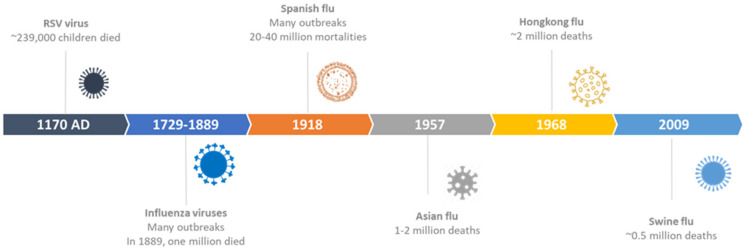
Pandemics caused by some of the respiratory tract-associated viruses and resulting mortalities.

Among various infections, acute respiratory tract infections (ARTIs) are the most common diseases affecting all individuals irrespective of age or gender [17]. These diseases are typically caused by various microorganisms including a variety of bacteria and viruses, such as *Streptococcus pneumoniae*, *Haemophilus influenzae*, *Moraxella catarrhalis,* Influenza A or B (“the flu”), respiratory syncytial virus (RSV), parainfluenza, adenovirus, coronaviruses, and others [18,19]. However, in terms of contagious ability and medical emergencies, the greatest infections are typically associated with RSV, Influenza A or B, and coronaviruses, which have caused several epidemics and pandemics [20]. Still, among others, the corona- and influenza viruses undoubtedly cause more serious symptoms. Indeed, some of the most serious and prolonged historical and current infection outbreaks are associated with these viruses which have affected a large population, but especially older individuals [21]. The word “Pandemic” has been taken from Greek and means “of all the people”—it typically refers to the widespread emergence of a disease over one or several regions of the world [22], while the local occurrence of infections is called an epidemic, which is often driven by seasonal strains of viruses. Typically, pandemics may arise when novel strains of viruses infect humans and promote human-to-human transmission before humans develop appreciable immunity to fight against these strains. Pandemics occur due to various genetic mechanisms, have unpredictable patterns of mortalities among individuals of all age groups, and differ vastly in how and when they emerge and recur. Pandemics caused by some of the viruses associated with respiratory tract are discussed below (Figure 1). 

**(A) RSV Virus:** Respiratory syncytial virus (RSV) infection is typically associated with recurrent wheezing and exacerbated lung disease. It is a major cause of hospitalization in infants and young children around the world and is also responsible for different epidemics [23]. RSV is a member of the pneumovirus genus of the family *Paramyxoviridae*. The virus contains an encapsidated non-segmented negative-sense RNA genome and a lipid envelope [24]. Historical evidence of RSV-associated infection exists dating back to 1170 AD in which Maimonides made the first observation and described the relation between bronchiolitis and recurrent wheezing. He wrote “I conclude that this disorder (asthma) starts with a common cold, especially in the rainy season, and that the patient is forced to gasp for breath day and night” [25]. RSV-based illnesses are recurring infections which mostly appear in the winter season in mild climates and also during rainy seasons in tropical regions. Every year, RSV-based epidemics exist for 4 to 5 months, which usually begin in autumn and last until late winter or early spring. The spread of infection is typically high in under-developed countries, where 97% of hospital admissions and 99% of total deaths are caused by RSV [26]. An extensive review of mortality rates in children younger than five years in 187 countries has revealed fatalities of ~239,000 children per year due to RSV infection [27]. Besides, epidemiological evidence also indicated the impact of RSV on the elderly, both in the community and in long-term care facilities [28]. The first reports of RSV infections in nursing home residents appeared in the 1970s, and since then, there have been at least 20 published accounts of RSV in long-term care facilities [29]. Some reports have also described RSV outbreaks involving between 8 and 52 residents. In another instance, a research study conducted in winter in a large nursing home (Rochester, NY, USA) has documented RSV infection in 40 persons; the overall attack rate was 7% and RSV was identified as the cause of 27% of the illnesses [30]. 

**(B) Influenza Virus:** The name *“influenza”* originates from Latin word “*influentia*” or “influence”, and the condition is caused by various genera of viruses belonging to the family *Orthomyxoviridae*. The genome is segmented RNA consisting of seven or eight segments. These viruses can be divided into A, B, and C types. Although all these strains are responsible for significant worldwide mortalities, only the type A influenza strain with an animal reservoir has the potential to cause deadly pandemics [31,32]. Typically, aquatic wildfowls are known to be endemic reservoirs for the influenza viruses, and most of these birds are migratory in nature. When these viruses are transferred from birds to mammalian species under various circumstances, they exist in their new host for longer time periods (even for several decades). Researchers have indicated the presence of influenza A virus in at least 18 mammalian species [33]. Different strains of influenza viruses have caused several human pandemics for centuries, which have resulted in extensive deaths and disruptions [34]. Indeed, in many cases, the word pandemic has been specifically used to refer the outbreaks of influenza viruses that contain novel hemagglutinin (HA) genes [35]. Notably, medical historians have mentioned various Greek writings from 412 BC which may have recorded the outbreak of various infections with symptoms similar to influenza [36]. However, due to the lack of technology to correctly identify the viruses until 20th century, historians could not directly associate the outbreaks with influenza virus. 

However, the reliable history of pandemics can only be documented after the first reference to “influenza” in scientific literature in 1650 [37]. In the eighteenth century, the first influenza pandemic was reported in Russia during the winter of 1729; from there, it spread across Europe over a period of six months and around the world in three years. The infection occurred in two different waves and caused significant mortalities [38]. The second pandemic began in the 18th century in Southeast Asia, which then spread to Russia and east Europe. It lasted for eight months and was known for extremely high infection rates in young individuals, but mortalities were negligible [39]. Similarly, in the 19th century, several pandemics occurred, including a major pandemic in the winter of 1830 which also started from Southeast Asia and spread through Russia and Europe to rest of the world over a period of one year. Although the rate of the infection was significantly high, the mortality remained low. In the same century, in 1889, one more pandemic occurred in Russia, which spread across Europe and North America [40]. The estimated fatalities in this infection were in the range of 0.1–0.28% and the pandemic killed ~1 million people globally [41]. 

In recent history (around 100 years), there have been four pandemics which have caused great disruption in the world and of which novel strains of influenza viruses have emerged as the causative agent, against which humans had little or no immunity. For instance, in 1918, the Spanish flu (H1N1); in 1957, the Asian flu (H2N2); in 1968, the Hong Kong flu (H3N2); and in 2009, the swine flu (H1N1) [42]. 

**(C) The Spanish Flu (1918):** One of the deadliest pandemics, “the Spanish flu”, occurred in 1918 and was caused by a novel strain of influenza virus, namely H1N1, which is often referred to as the ‘greatest medical holocaust in history’ [43]. It started in the autumn of 1918 in France where thousands of soldiers died. The disease then quickly spread to other parts of the world and ended up killing between 20 and 40 million people in just 8 months [44]. Besides, several waves of the pandemic have also occurred (the timing and number is not consistent), including three specific waves in the spring of 1918, the fall of 1918, and the winter of 1918–1919 [45]. Among these waves, the second wave in the fall of 1918 was the deadliest, which resulted in the deaths of millions of people. Despite insufficient data, it is widely believed that the virus infected almost half of the world’s population and caused significant social and economic disruptions due to the shutdowns of schools and businesses [46]. Unlike other viral epidemics, the Spanish flu had mostly affected healthy individuals among the younger population between the ages of 18 and 40 years, possibly due to the excessive immune response, also known as cytokine storm, which made young individuals more vulnerable due to their strong immune system [47].

The origin of the Spanish flu is still debated; however, it is highly unlikely that it originated in Spain as the name suggests, which came into existence due to the result of a widespread misunderstanding [48]. Spain was among the few European countries which remained neutral during the First World War; therefore, the Spanish media was not censored and was free to report about the sickness in much detail in late May 1918. Since people around the world got to know about the disease in detail from Spanish media, they probably assumed Spain to be the origin of the infection. Still, there is no consensus among researchers about the actual origin of the disease; nevertheless, China, France, and Britain have all been suggested as the potential birthplace of the virus. Besides, the United States is also considered among the list, where the first known case was reported at a military base in Kansas on 11 March 1918 [49]. Recently, a detailed investigation about the emergence of virulent respiratory diseases before the outbreak of Spanish flu has suggested that the origin of the first wave of Spanish flu was an outbreak in China that was misidentified as pneumonic plague. From there it spread over the world through the Chinese Labor Corps (CLC), a group that, from 1916 to the end of the First World War, sent over 100,000 laborers to Europe to support the Allied war effort [50]. 

Initially, the Spanish flu was believed to be caused by the bacterium *Haemophilus influenzae*, formerly known as *Bacillus influenzae*, although physicians could not find any *bacilli* during autopsies [51]. Due to limitations such as lack of knowledge and technology, it was difficult to diagnose the influenza infections. Besides, it was often mistaken with common cold, cholera, or bubonic plague. It was later recognized that the transmission occurred by infectious respiratory droplets from the nose and throat. Nevertheless, the disease was largely ignored in the early stages and was not contained until it was already widely spread [46]. To make the matter worst, in that period, antibiotics and effective vaccines were not available to treat or prevent influenza and secondary bacterial infections. Therefore, efforts to control outbreaks largely relied on non-pharmaceutical interventions (NPIs) such as quarantines, school closures, banning public gatherings, and infection prevention practices such as minimizing the spread through cough and sneeze by using facemasks [52]. 

**(D) The Asian Flu (1957):** After the Spanish flu, which was caused by H1N1 influenza virus, a novel strain of the same virus, which was named as H2N2, emerged in the month of February in 1957 in the Yunnan Province of China [53]. The pandemic originated in Southeast Asia, and before spreading globally, it travelled to Hong Kong in April, then to Singapore, Taiwan, and Japan. The virus mostly affected individuals over 65 years due to the lack of immunity to novel strains. However, its mortality rate was relatively much lower compared to the other two pandemics that occurred in 1889 and 1918 [54]. Like many other infections, the Asian flu also appeared in successive waves and contained a more severe second wave than the first one. The infection was relatively mild with an estimated fatality rate of ~0.67% and caused around 1–2 million deaths [55]. This low fatality rate can also be due to the significant improvement in scientific knowledge and advancement in technology for the detection and control of viruses in the post-Spanish-flu era. During this period, a large network of laboratories developed throughout the countries which were connected to the Influenza Research Center based in London, which allowed to study the strain soon after it emerged [56]. However, the process of vaccine development was rather slow and it took moths to distribute the vaccine to high-risk individuals and personnel associated with essential services. Various approaches were adopted to treat the infected individuals, which included the prescription of antibiotics to seriously sick patients. However, development of antivirals remained elusive even at that time. Use of antibiotics prevented the spread of secondary bacterial infections, but they alone were not effective in treating the viral infections. 

**(E) The Hong Kong Flu (1968):** After the Asian flu, the virus underwent a significant shift and was named as a H3N2 type virus, which caused a new pandemic known as the Hong Kong flu in 1968. Influenza virus continued to infect human beings due to the high frequency of changes in the antigenic sites of the virus. Two distinct surface antigens of influenza virus, hemagglutinin (HA) and neuraminidase (NA), both undergo a gradual antigenic drift. However, the significant drift in the HA antigen is mainly responsible for the influenza epidemics of varying impacts [57]. However, the abrupt and complete antigenic shift of both HA and NA may cause a deadly pandemic which may occur occasionally. For instance, in the influenza pandemic of the Asian flu (1957), both antigens HA and NA changed, whereas in the case of the Hong Kong flu (1968), only the former antigen (HA) changed. Although the Hong Kong flu was milder than the Asian flu, it spread at a much faster pace due to the extensive air travel during that period [58]. When the excess all-cause mortalities were compared (although the figures may not be accurate due to the variations in calculating methods, they may provide valuable approximation), Spanish flu caused 598 deaths per 100,000 individuals, whereas the Asian flu and the Hong Kong flu have caused only 40.6 and 16.9 deaths, respectively [35]. During this pandemic, estimated deaths in the range of 500,000 to two million have been recorded, which is approximately similar to the deaths recorded during the Asian flu. Originating in Hong Kong in 1968, the virus quickly spread to the USA through the US army soldiers returning after the Vietnam War. Subsequently, it spread to other parts of the world including Japan, England, Australia, Canada, France, etc. This virus also exhibited high mortality towards younger individuals, as the highest death rates were reported among children [59]. During the pandemic, routine infection control methods were implemented, and other methods such as vaccination, intensive care for complicated cases, and administration of antibiotics to treat secondary infection were also used. 

**Figure 2:**
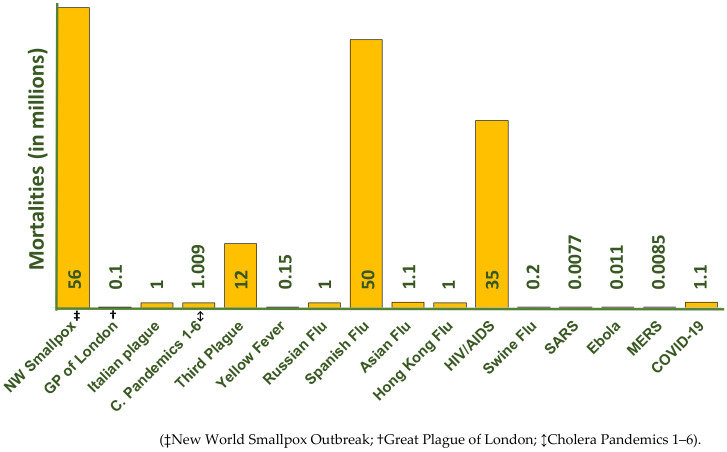
World pandemic history.

**(F) The Swine Flu (2009):** In April 2009, another outbreak of disease was recorded simultaneously in Mexico and the United States which was also related to influenza A virus of the type pH1N1/09. This was identified as the etiologic agent of most swine influenza cases and the disease was called the Mexican flu or New flu [60]. This virus is known to have originated in pigs, and the outbreak was caused by the triple viral re-assortment between two influenza lineages that had been circulating in pigs for a long time. This outbreak was also declared as a pandemic in June 2009, which quickly spread across 30 countries in a few weeks [61]. Unlike the past pandemics, which took an average of 6 months for spreading, the swine flu spread very quickly in weeks, as initially, no restrictions were placed on trade and travel. Consequently, in the next few months, the infection spread across 122 countries, infecting 134,000 people, and caused almost 800 deaths [55]. However, the figures were later revised based on new mathematical models. These models also found that the deaths recorded previously were often under-reported [62]. In this case, the mortality was assumed to be somewhere between 151,700 and 575,400 [63]. Like other pandemics of 20th century, the swine flu has also exhibited wave behavior; however, the time and number of waves varied depending on the countries. For example, in Mexico and India, three waves were recorded, whereas in Europe and Northern America, the infection occurred in two different waves [64,65]. This pandemic also resulted in high mortality in younger individuals, especially children and pregnant women as reported in the previous outbreaks. However, unlike in previous pandemics, an enhanced level of preparedness was witnessed across the globe during this outbreak. Since the medical fraternity was on high alert due to the fear of fresh outbreaks of viruses after the spread of SARS and H5N1 avian flu. Mostly, in this case, non-pharmaceutical measures were also applied, such as recommendation of hand hygiene and voluntary isolation of infected individuals, etc. Mortalities due the various pandemics are given in Figure 2. 

## 3. Coronaviruses and Earlier Outbreaks

**Figure 3:**
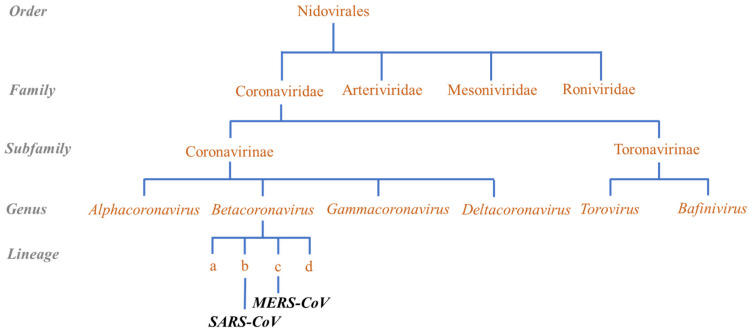
Classification scheme of coronaviruses.

The latest outbreak of another ARTI (acute respiratory tract infection), COVID-19, has once again brought the attention of the world towards the deadly viruses and tested our capability of dealing with the threat of highly contagious viruses including coronaviruses which are a known health threat [66]. Coronavirus has been known to cause human infections since the 1960s; however, the potential of this virus to cause deadly epidemics came to fore in the last two decades only. COVID-19 is the third major outbreak of respiratory disease in twenty years related to coronavirus, which has significantly disturbed the socioeconomic balance of the entire world. SARS-CoV-2 belongs to the family *Coronaviridae,* which belongs to the order *Nidovirales* [67]. The family contains two subfamilies, *Coronavirinae* and *Torovirinae*. *Coronavirinae* are classified into four genera: *Alphacoronavirus*, *Betacoronavirus*, *Gammacoronavirus*, and *Deltacoronavirus*. Earlier, the genus *Betacoronavirus* was subdivided into lineages A, B, C, and D. Now, these lineages have been classified as subgenera of *Betacoronavirus*—as *Embecovirus* (lineage A), *Sarbecovirus* (lineage B), *Merbecovirus* (lineage C), and *Nobecovirus* (lineage D) (Figure 3). SARS-CoV-2 belongs to the genus *Betacoronavirus* and subgenus *Sarbecovirus*. Coronaviruses are enveloped round-shaped viruses, and sometimes they are pleiomorphic, of approximately 80 to 120 nm in diameter. The virus is characterized by the presence of club-shaped spike projections originating from the surface of the virus [68]. These spikes are responsible for their typical appearance similar to a solar corona, giving it the name coronavirus. Coronaviruses are heat- and ultraviolet ray-sensitive but can be stored for many years at a temperature of −80 °C. However, these viruses can be inactivated at 56 °C for 30 min, which is often carried out by researchers. In addition, chlorine-containing disinfectants, peracetic acid, and 75% ethanol can also deactivate coronaviruses [10].

Being a zoonotic virus, coronavirus has the ability to transmit from animal to humans and also among humans through airborne aerosols [69]. So far, several animals and birds have been identified as reservoirs for this virus, including camels, pigs, turkey, mice, dogs, bats, cats, and others. However, among these animals, the bat is the most widely known carrier for human infections [70,71]. Initial cases of coronavirus infection in humans were reported in 1960, which was assumed to be a reason for common cold. However, the potential of coronavirus as a cause of respiratory illness was known much later. Prior to the outbreak of 2002 SARS-CoV, different subtypes of coronaviruses were reported to infect human beings which were responsible for causing mild respiratory infections [72]. These include two *Alphacoronavirus* species, HCoV-229E and HCoV-NL63, and two *Betacoronavirus* species, HCoV-OC43 and HCoV-HKU1 [68]. For instance, Kaye et al. have examined acute and convalescent serum pairs and control sera collected from subjects living in a children’s home over a 7-year period (1960–1967) using the hemagglutination inhibition (HI) test with the coronavirus strain HCoV-OC43 [73]. During the study of 93 serologic conversions, 44 were related with reported illnesses and 49 with no reported illnesses. A total of 67 conversions occurred in three different outbreaks during the winter and spring quarter of 1960–1961, 1964–1965, and 1966–1967. It was revealed that during the period of seven years, the OC43 strain of *β*-coronavirus was responsible for 3% of the total 1328 respiratory illnesses. However, in 2002, the world witnessed the first lethal coronavirus-induced disease which was named as severe acute respiratory syndrome (SARS-CoV). A decade later in 2012, another outbreak of coronavirus infection was reported in Saudi Arabia, which is known as Middle Eastern respiratory syndrome (MERS-CoV).

SARS-CoV and MERS-CoV belong to the genus *Betacoronavirus* in the *Coronaviridae* family and have large, positive-sense RNA genomes of 27.9 and 30.1 kb, respectively [74]. Mostly, bats are the reservoir of a large variety of coronaviruses, including SARS-CoV- and MERS-CoV-like viruses [75]. Human-to-human transmission of SARS-CoV and MERS-CoV occurs mainly through nosocomial transmission; 43.5–100% of MERS cases in individual outbreaks were linked to hospitals, and very similar observations were made for some of the SARS clusters. The clinical courses of SARS and MERS are remarkably similar, except for a few differences. Although the pathogenesis of MERS is poorly understood, similar mechanisms may underlie the pathogenesis of both MERS and SARS [76].

The first known case of atypical pneumonia associated with SARS-CoV was reported in Foshan, China, in the month of November 2002 [77]. Since then, the outbreak of the disease started to spread quickly across the globe, which prompted the World Health Organization (WHO) to declare the ailment “a worldwide health threat”. After the emergence of the disease in mainland China, within a few months, >300 cases were reported; among these cases, the majority of them were healthcare workers. Subsequently, the traveling of infected individuals further spread the disease to other countries including Hong Kong, Vietnam, Canada, and many more [78]. To deal with this outbreak, in March 2003, the WHO coordinated with a large network of research centers around the world to identify the causative agent of SARS. In one such effort, Drosten et al. investigated the patients affected by the disease in the same year and they revealed that a novel form of coronavirus might have caused SARS [79]. However, the genetic characterization, which confirmed the novelty of the virus, indicated that the virus is only distantly related to known coronaviruses (only identical in 50 to 60 percent of the nucleotide sequence). The SARS pandemic not only caused a public health problem but also initiated socio-economic crises, particularly in China [80]. In the beginning, it was believed that the pandemic may spread globally and cause serious economic downturn. However, the prompt actions taken by the authorities dealing with the epidemic such as isolating suspects, contact tracing, and quarantine measures served well to effectively contain the illness [81]. Subsequently, the SARS pandemic ended in July 2003, but before that, it infected 8096 individuals and caused 774 deaths across 27 countries [82]. A few more cases of SARS were again reported at the end of 2003 (December–January 2004) due to zoonotic transmission, possibly involving civet cat (*Paguma larvata*). However, since then, no more human cases associated with SARS have been detected [83]. Although SARS had low mortality and morbidity, the health consequences of the SARS pandemic were not limited to people who were infected. Indeed, it fueled great fear among the general population due to the novelty of the causative virus, the ability of rapid nosocomial transmission, and the vulnerability of hospitals and healthcare workers [84].

A decade after the occurrence of SARS-CoV, a case of acute pneumonia and renal fever was reported in June 2012 in Saudi Arabia. The death was associated with another novel form of coronavirus, MERS-CoV (Middle East respiratory syndrome coronavirus), which was isolated from the sputum of the patient [85]. However, prior to the discovery of first MERS case in Saudi Arabia, an outbreak of acute respiratory illness emerged in a public hospital in Zarqa, Jordan, in April 2012 [86]. Eleven individuals were found to be affected, which included eight healthcare workers, and one of them died later. At the time of the outbreak, the cause of the disease was unknown since the epidemiological investigation including laboratory testing carried out after the emergence of disease remained inconclusive. However, after the discovery of the novel coronavirus infection in the Arabian Peninsula, stored respiratory and serum samples of patients from this outbreak were retested and the diagnosis of MERS-CoV was confirmed in two deceased patients. Thereafter, a few more cases were reported in the UK and the disease continued to spread to other parts of the world through the traveling of infected individuals. Most of the imported MERS cases were reported due to nosocomial transmission. In May 2015, the first MERS patient was confirmed in South Korea who returned from the Middle East. As of July 26, within approximately two months, 186 cases were confirmed, including 36 deaths and 138 recoveries [87]. The MERS outbreak in Korea was characterized by intra-hospital transmission as well as hospital-to-hospital transmission due to the movement of cases from one hospital to another. It was the largest MERS outbreak outside the Middle East, involving 16 hospital-acquired infections. According to the WHO data, a total of 2494 laboratory-confirmed cases of MERS have been reported as of November 2019, including 858 deaths in 27 countries (WHO MERS). In this illness, symptoms such as fever, cough, and shortness of breath have been reported. Although pneumonia was commonly reported, it was not always present. Besides, gastrointestinal symptoms, including diarrhea, were also reported. Notably, some laboratory-confirmed cases of MERS-CoV infection have been reported to be asymptomatic, which means some individuals did not exhibit any clinical symptoms but they still tested positive for MERS-CoV infection. Most of these asymptomatic cases were detected following aggressive contact tracing of a laboratory-confirmed case [88]. In most of the cases, infection spread via human-to-human transmission in healthcare settings. However, a few studies have suggested the role of dromedary camels as reservoir hosts for MERS-CoV and as an animal source of MERS infection in humans. However, the exact role of dromedaries in transmission of the virus and the exact route(s) of transmission are unknown [89]. During the outbreak of SARS-CoV, medical and scientific communities were not sufficiently prepared for dealing the threat of a deadly pathogenic virus. However, a decade later, healthcare professionals and researchers were relatively better prepared when the MERS-CoV pandemic emerged due to the advancement in molecular diagnostic tools such as the availability of advanced sequencing tools and next-generation sequencing technologies that made full-length genome sequencing easier. 

## 4. What Is New about COVID-19

**Figure 4:**
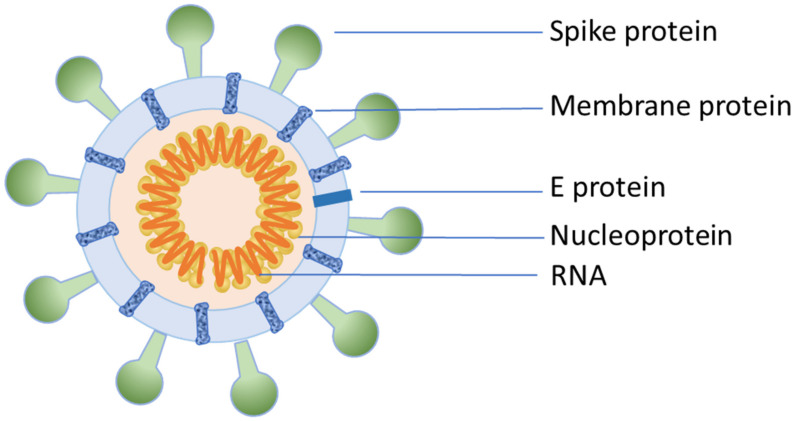
Typical scheme of severe acute respiratory syndrome coronavirus 2 (SARS-CoV-2) virion structure.

The emergence of a new pandemic in December 2019 associated with an old viral threat “coronavirus” has once again revealed the vulnerability of world in handling such a massive pandemic. After the outbreak of SARS-CoV and MERS-CoV, COVID-19 is the third major coronavirus outbreak, which proved to be the deadliest among all the previous outbreaks. SARS-CoV-2 is a novel form of coronavirus belonging to the genus *Betacoronavirus* and subgenus *Sarbecovirus*, while the species is known as severe acute respiratory syndrome-related coronavirus species. The virus is ellipsoidal in shape, with an average diameter of 64.8 ± 11.8, 85.9 ± 9.4, and 96.6 ± 11.8 nm (average ± SD) for the short, medium, and long axis of the envelope, respectively, and has a characteristic crown-shaped appearance [90,91] (Figure 4). Spike proteins give the characteristic appearance to the virus and the copy number of spike proteins is 10-times higher than the influenza virus and is comparable to HIV. Spikes can rotate along the stalk freely. A minor population of the virus shows Y-shaped spiked pairs with two heads and one stem. Its RNA is packed with ribonucleoproteins (RNPs) in the lumen of the virus, with each particle containing about 30–35 RNPs. 

SARS-CoV-2 contains a single-stranded positive-sense RNA genome that is 29.8 to 29.9 kilobases in length, which is packed in ~80-nm-diameter lumen and contains fourteen open reading frames [92,93]. The genome encodes sixteen non-structural proteins involved in viral replication and transcription such as RNA-dependent RNA polymerase and various structural proteins such as the spike surface glycoprotein, nucleocapsid protein, and envelope and matrix proteins. Researchers have indicated that SARS-CoV-2 also belongs to the same species as SARS-CoV. However, it is different from both zoonotic coronavirus MERS-CoV and SARS-CoV introduced to humans earlier in the twenty-first century and that is why it is also called as novel coronavirus or SARS-CoV-2. The comparison of the genome shows that SARS-CoV-2 has only 79.5% and 40% homology with SARS-CoV and MERS-CoV, respectively. Comparison between SARS and SARS-CoV-2 genomes has shown that there are 380 amino acid substitutions between SARS-CoV-2 and SARS-like coronaviruses. Twenty-seven mutations are involved in spike proteins (S proteins) [76]. This may be responsible for the changed infection rates of the virus. The outer subdomain of the SARS-CoV-2 S protein which binds to receptor has only 40% amino acid homology with other SARS-associated coronaviruses [10]. In a phylogenetic tree, SARS-CoV and SARS-CoV-2 form a single clade which is different from MERS-CoV [94].

Notably, the symptoms, epidemiology, incubation period, and radiological findings of COVID-19 patients are almost similar to those of SARS patients. Typically, infections associated with coronavirus occur by the involvement of receptors on the surface of the host cell membrane. Initially it was thought that SARS-CoV enters the host cell through direct fusion with the plasma membrane, but later it was found that the virus gains entry into the cell through pH- and receptor-mediated endocytosis [95]. Coronavirus invades the host cell through a process of clathrin-mediated endocytosis, during which the S proteins (spike glycoproteins) present on the surface of the coronavirus recognize and bind to the receptors on the host cell [95]. These structural proteins are essential for the assembly and infection of coronavirus, which consists of S1 and S2 subunits. The S1 subunit contains the receptor-binding domain (RBD) and binds to the cellular receptor and the S2 subunit facilitates the fusion and entrance process [96]. However, different types of coronaviruses utilize different cell receptors to invade the host cells [97]. For instance, HCoV-229E uses aminopeptidase N as a receptor, whereas SARS-CoV and MERS-CoV utilize ACE2 and DPP4 (CD26) receptors, respectively. On the other hand, COVID-19 uses angiotensin converting enzyme II (ACE2) receptors to bind to the surface of host cells such as SARS-CoV [98]. Unlike other forms of coronavirus which exhibited low pathogenicity (showed mild respiratory symptoms), SARS-CoV, MERS-CoV, and SARS-CoV-2 have caused serious outbreak of illness with acute symptoms and high mortality. Particularly, the intense infection associated with SARS-CoV-2 is attributed to the hyper-activation of T cells as revealed by pathological findings. The acute immune injury of COVID-19 patients is caused by the considerable enhancement of Th17 cells and lower count and high cytotoxicity of CD8 T cells [99]. 

## 5. Mode of Infection

Coronaviruses, typically, are enveloped viruses with a single-strand, positive-sense RNA genome with a size of ~26 to 32 kilobases, which is the largest known genome for an RNA virus [100]. Although all types of coronaviruses share similarities in the organization and expression of their genome, based on their phylogeny, they have been classified into four different genera: *Alphacoronavirus*, *Betacoronavirus*, *Gammacoronavirus*, and *Deltacoronavirus*. The S glycoprotein binds to the ACE2 receptor on the surface of host to gain entry into the cell. The S_1_ subunit of the *Betacoronavirus* spike proteins displays a multi-domain architecture and is structurally organized in four distinct domains: A–D. Among these domains, A and B possibly serve as receptor-binding domains (RBDs), necessary for binding with host cell receptor. Meanwhile, the S2 subunit contains other domains required for fusion and intracellular trafficking into the host cell. The S glycoprotein binds with the ACE2 (an ectoenzyme) receptor on the surface of host cell. Other studies have elaborated that the S glycoprotein is cleaved at specific sites by host proteases furin before it can bind to the receptor [101].

Different types of coronavirus infections are commonly found in various domestic animals, such as respiratory disease in dogs (canine respiratory coronavirus), gastrointestinal infections in bovine, canine, turkey, etc., which are typically caused by a variety of coronaviruses including bovine coronavirus, feline coronavirus, etc. [102], whereas coronavirus infections in human beings typically affect the upper respiratory and gastrointestinal tracts and may often cause mild, self-limiting diseases including common cold; however, in many cases, it is also responsible for more severe manifestations such as bronchitis and acute pneumonia [103]. Currently, there are seven different types of coronaviruses which are known to cause infections in humans; these are classified into low pathogenicity (HCoV 229E, NL63, OC43, and HKU1) and high pathogenicity coronaviruses (SARS-CoV, MERS-CoV, and SARS-CoV-2). Low pathogenicity coronaviruses typically cause mild diseases with non-serious respiratory syndrome, which are globally endemic. However, in the last two decades, three highly pathogenic, novel zoonotic coronaviruses have emerged and have caused lethal human infections, including SARS-CoV-2 which has generated grave public concern.

## 6. Treatment of COVID-19 and Vaccine

**(A) Drugs:** Although to date, there is no drug to successfully treat COVID-19, scientists have shown some success of broad-spectrum antivirals and some other drugs in treating the infections of SARS-CoV-2. Around 15 different drugs are being tested for the treatment of COVID-19 infections. These include, chloroquine and hydroxychloroquine, lopinavir and ritonavir, nafamostat and camostat, famotidine, umifenovir, nitazoxanide, ivermectin, corticosteroids, tocilizumab and sarilumab, bevacizumab, and fluvoxamine [104]. Various antiviral agents such as remdesivir and ribavirin were tested for their efficacy in treating the disease. For example, remdesivir (GS-5734) has a broad-spectrum activity and its activity against MERS and SARS has been demonstrated in animal trials [105,106,107]. Remdesivir is known to inhibit the activity of RNA-dependent RNA polymerase activity, therefore inhibiting transcription of viral RNA. Some trials of remdesivir to treat COVID-19 were also carried out, but it was found to have some adverse effects [105,106,107]. Another widely used antiviral drug is ribavirin, known to inhibit the synthesis of ribonucleoprotein. It also inhibits the early transcription of viral genes and, therefore, is known to inhibit the replication and spread of the virus [108]. The trials of ribavirin remain inconclusive, as some studies show no effect of the drug on COVID-19 patients [109]. Earlier, the US FDA clearly stated that this drug is not effective for the treatment of influenza. Similarly, other antiviral agents including lopinavir and ritonavir are also being tested to treat COVID-19 patients, as these are known to have positive effects on MERS and SARS-CoV-1 patients [91]. Some of the studies suggested that chloroquine and its derivatives can inhibit replication of the virus in vitro [110]. Some of the possible mechanisms of action involve reducing endosomal pH which will result in degradation of viral proteins, and interference with terminal glycosylation of the cellular receptor ACE2, minimizing the binding of the virus [111]. It is also documented that the drug may also interfere with angiotensin-converting enzyme 2, which is one of the binding sites for SARS-CoV S proteins [112]. However, recently published studies about the trials carried out throughout the world do not show any positive impact of the drug [113,114]. The most important problem with COVID-19 infections is the acute respiratory distress syndrome (ARDS). Corticosteroids can be used for offsetting the ARDS by offsetting the cytokine storm. However, this immunomodulatory effect also exposes patients to secondary infections and other complications [115].

**(B) Support therapy/management:** Since there is no effective medicine for the treatment of COVID-19, the disease results in a number of complications in the patients. This will require a number of life support therapies to endure and minimize the losses caused by the disease. These include therapies such as artificial liver system (ALS) and extracorporeal membrane oxygenation (ECMO). It is clear from various trials and other studies that there is no specific treatment for COVID-19 and the patients are being treated using combinations of different medicines and management practices [116]. According to the WHO, there are more than a dozen vaccines in clinical phase 3 trials developed by various organizations and research groups; these include the vaccine developed by the University of Oxford/AstraZeneca [117]. Unfortunately, the trials were stopped due to some side effects in some of the patients. Other vaccines under clinical phase 3 trials include the vaccines developed by CanSino Biological Inc./Beijing Institute of Biotechnology, Sinovac, Moderna/NIAID, and many others [118]. These are mainly non-replicating viral vector or inactivated viruses [119]. Very recently, the Pfizer-BioNTech COVID-19 vaccine has been authorized and recommended. Other vaccines that have been developed include Moderna’s COVID-19 vaccine, Sinovac, and Sputnik V.

## 7. Epidemiology of COVID-19

Since SARS-CoV, MERS-CoV, and SARS-CoV-2 are not well adapted to be maintained in humans, they are likely to spread mainly through other zoonotic reservoir(s), with occasional outbreak in the susceptible human population, possibly via an intermediate host species [120]. Particularly, the human-to-human transmission rate of the novel coronavirus is significantly high, which causes a wide spectrum of clinical manifestations in patients infected with virus [121]. For instance, Guan et al. have performed a detail analysis of the clinical characteristics of affected patients from 552 hospitals from 30 provinces in China, just after a month of emergence of the disease. Out of 1099 patients with laboratory-confirmed COVID-19 cases, ~48% of the patients were male. Initially, the diagnosis of the illness was difficult due to the existence of diverse symptoms. Among the patients studied, 43.8% exhibited fever at the time of presentation, but after hospitalization, the figure increased to 88.7%. About 15.7% of the patients developed serious symptoms after admission to a hospital [122]. Despite the huge number of deaths associated with COVID-19, SARS-CoV-2 appears to have a lower fatality rate when compared to SARS-CoV or MERS-CoV. The rapid spread of disease has prompted public health official and government bodies to enforce unprecedented measures such as travel restrictions, imposing large-scale curfews, isolation and quarantine of infected individuals, etc. 

Since the outbreak of COVID-19 in December in Wuhan, China, the infection has rapidly spread to other parts of the world, and the growing number of cases clearly suggests that the illness is still continuously spreading. Initially, several cases (>50 people) of acute pneumonia associated with COVID-19 were reported in China, which were linked to a seafood market in the Wuhan province. Since then, the number of infected individuals has reached around ten million, which may still be an underestimate since there is a strong possibility of untraced exposures and asymptotic individuals. Sequence-based analysis of isolates from patients has led to the identification of the causative agent as a novel form of coronavirus. Besides, sequencing technology and other techniques have significantly helped in correct diagnosis of the viral infection [123]. Initially, individuals who had visited the seafood market or consumed the food prepared with the infected animals have been supposed to be infected with SARS-CoV-2. Later on, further analysis and contact tracing of COVID-19-positive patients revealed that a number of individuals with no history of travelling to the seafood market were also tested positive for the COVID-19 disease. These results have pointed towards the possibility of a human-to-human transmission of the virus, which was subsequently reported in more than 200 countries around the world.

The possibility of human-to-human transmission of SARS-CoV-2 was confirmed in an epidemiological study of patients in a family cluster, in which some of the members visited Wuhan, but one member of the family did not visit the place [124]. In another study, Liu et al. reported the emergence of SARS-CoV-2 infection at an epidemic level in Shenzhen, China [125]. This large-scale study has indicated the possibility of community transmission and intra-family transmission as the main reasons of SARS-CoV-2 spread in the city. The human-to-human transmission of SARS-CoV-2 mostly happens in the presence of the close proximity of an infected individual due to the exposure to cough, sneeze, respiratory droplets, or aerosols. These aerosols may reach lungs via inhalation through the oral or nasal passage. Similar to other respiratory infections such as SARS-CoV and MERS-CoV, SARS-CoV-2 is transmitted through droplets of various sizes. Typically, droplets with particle diameter of more than 5 to 10 μm are considered as respiratory droplets, whereas droplets with diameter of less than 5 μm are referred to as nuclei [126]. The transmission of diseases by nuclei droplets containing the virus, which remained after the evaporation of large droplets, is usually referred to as airborne transmission. These airborne droplets remain in the atmosphere for a very long time and can be transmitted among individuals standing at large distance of more than a meter. On the other hand, SARS-CoV-2 is mostly transmitted through respiratory droplets and contact routes. Indeed, in a detailed analysis of over 75,000 patients with COVID in China, indication of airborne transmission was not reported [127]. 

Droplet transmission of SARS-CoV-2 happens when a person is in close contact of <1 m with an individual suffering from respiratory symptoms such as cough or sneezing. The infected individual can potentially transmit the virus through the infected droplets of his/her mucosae (mouth and nose) or conjunctiva (eyes). Although transmission through mucosae is most common, the transmission of the virus through conjunctiva is relatively less common [128]. In addition, similar to the other coronaviruses, SARS-CoV-2 is also potentially responsible for nosocomial outbreak through environmental contamination as a route of transmission. However, the mode and extent of environmental contamination still need to be investigated. Recently, Ong et al. have studied different fomites including surface samples of objects used on infected individuals, PPE samples, and swabs, etc., from the patients lodged in a well-protected isolation room (12 air exchanges per hour). The study has suggested environmental contamination by SARS-CoV-2-positive patients through respiratory droplets and fecal shedding to be potential threats for the transmission of disease [129]. 

## 8. How COVID-19 Became a Pandemic

The origin of COVID-19 is believed to be a seafood wholesale market in Hunan which also sells different types of wild animals including snakes, birds, bats, rabbits, and frogs, etc. The sequence analysis of various species of coronavirus revealed that SARS-CoV-2 is a recombinant virus between the bat coronavirus and a coronavirus of an unknown source, which is suspected to be the pangolin [130]. After the official declaration about the novel SARS-CoV-2 as a potential cause of the COVID-19 outbreak in Wuhan, China, the most critical question in the minds of governments and public health officials was the possibility of SARS-CoV-2 causing a global pandemic [131] as a pandemic affects various aspects of healthcare systems and requires extensive planning regarding the arrangements of supplies, availability of human resources, and ensuring the sustainability of the health system through the peak and duration of the epidemic. Moreover, drastic measures were needed to contain the pandemic. For instance, implementation of strict social distancing and mobility restrictions, such as closures of schools, public offices, gardens, etc., and strict travel advisories/bans, which seriously disrupt social and economic stability.

After the report of 29 pneumonia cases of unknown etiology in a district of central China, within one week, the cases were attributed to the local seafood market of live poultry and wild animals. On 12th January, a novel form of *Betacoronavirus* was declared as the causative agent for the disease and the infection was named as COVID-19. The disease had already spread throughout China and also to some neighboring countries within one month. Indeed, by the end of January, some cases of COVID-19 had already been reported in Europe and USA. By this time, the highly contagious nature (particularly human-to-human transmission) of the virus was already known to the world; however, unlike earlier pandemics of coronaviruses, SARS-CoV-2 was considered less virulent, with a relatively much lower number of critically ill patients (less than 1% even by October 29, 2020). The infection was declared as an international public health emergency by the WHO on 30 January 2020 with the highest level of concern [13] At that time (30 January 2020), the disease had spread to almost 20 countries, and 10,000 laboratory-confirmed cases and 200 deaths were recorded. Particularly, in China, several measures were taken to fast-track the availability of effective tests, vaccines, and medicines that can be used to save lives and avert a large-scale crisis. The authorities conducted vigorous search operations to find active cases and steps were even taken to identify persons who came in close contact of infected individuals (contact tracing). Patients with serious conditions were hospitalized, and those with mild symptoms were home quarantined or sent to specialized isolation centers. In order to control the further spread of the disease, several restrictions were enforced, including closure of public places such as schools and parks, stopping international flights and local transport, people were strictly advised to stay home, etc., and gradually, the most affected province of Hubei with a population of more than 50 million people was quarantined. 

As of 10 March, >48,000 confirmed cases and ~3000 deaths were reported across the globe. Finally, the WHO declared COVID-19 as a pandemic on 11 March 2020 [132]. Notably, after 15 March, a sharp rise in the number of infected cases and death rate was observed, and by the end of March, the number of infected individuals increased to more than 640,000 and the death rate crossed >18% (all of these figures were taken from worldometers) [133]. Initially, the highest number of cases was reported in China; however, by mid-March, Europe had recorded a higher number of cases than anywhere in the world, while COVID cases had spread to more than 160 countries and territories involving six continents. So far, health ministries, public health universities, medical research centers, and other health agencies across the globe have been working tirelessly to minimize the threat of this massive pandemic. However, it has not only proven to be a medical emergency but will be considered as one of the greatest human tragedies after World War II. It has seriously affected economic activities and has had immense socio-economic effects and wide implications on global trade, travel and supply chains, and it considerably disturbed the daily lives of the people across the globe. 

## 9. Some Statistics of COVID-19 

In order to properly understand the pandemic, authentic and reliable data are essential, which potentially help to study the spread of disease and measure the impact of outbreak on the lives of people around the world. Besides, statistical data also help to evaluate the measures taken by different countries to contain the pandemic. Initially, the assessment of different aspects of disease, including spreading rate, death rate, etc., was highly uncertain due to the limited availability of data and difficulties in accurate diagnosis. However, with accumulation of plenty of data and concerted efforts of various research agencies, more reliable inferences can now be made. Still, the total analysis of the COVID-19 pandemic is ambiguously reported at different levels. For example, the “number of COVID cases” reported by various media outlets is usually not precise. As in many cases, the patients showing symptoms were considered as confirmed cases without accurate laboratory testing, while a confirmed case is “a person with laboratory confirmation of SARS-CoV-2 infection”. Confirmed cases are therefore only a subset of the total number of cases. It is a count of only those people who have COVID-19 and for whom a lab has confirmed this diagnosis [134]. The number of confirmed cases is lower than the number of total cases because not everyone is tested. In this regard, most countries have struggled to test a large number of individuals who should have been tested, especially countries with a large population size. 

The next essential parameter in analyzing the COVID-19 data is the doubling rate, which essentially means how long it took to double the number of confirmed cases. During the spreading of an infectious disease, although the initial numbers look small, if the spread is not controlled, the growth of cases is rapid. For instance, countries such as the USA, Brazil, Russia, and India did not implement comparable measures; therefore, the numbers of cases have been rising fast. A detailed country-wise analysis of the count of COVID-19-related cases in the 10 countries with the most cases is given in Table 1 (Roser and Max et al. 2020). Based on the analysis of different types of data, scientists and health professionals have advocated several measures to prevent further spread of the disease. One of the most important measured suggested was mass testing as it is crucial to avoid and control spread of the infection and to quickly receive the care they need. 

## 10. Technical Problems in Containing the Virus

The rapid spread of infections through various means, including by asymptomatic carriers, has potentially transformed the COVID-19 illness from an epidemic to a global pandemic in a short period of time [135]. The numbers of cases through human-to-human transmission increased exponentially due to direct contact or through droplets [136]. Hospital-related transmission of SARS-CoV-2 contributed to ~41% of total cases [137]. Based on the evaluation of data collected from different parts of the world from the beginning of the disease in December 2020, the trend of increasing infections largely follows exponential growth, and the mean basic reproduction number (*R*_0_) was estimated to range from 1.4 to 4, as revealed by various studies [138,139]. R_0_ or ‘R-naught’ denotes the number of new infections estimated to emerge from a single case. This means if R_0_ is 2.5, then one person with the disease is expected to infect, on average, 2.5 others, and the doubling time of the disease is estimated to be 6.4 days, whereas R_0_ below 1 indicates a shrinking of number of infections [140,141]. The current estimate of the mean incubation period for the COVID-19 disease is 6.4 days, ranging from 2 to 14 days, with potential asymptomatic transmission, although the situation is evolving rapidly and there is great potential that the infection will continue to spread around the globe [142]. 

Therefore, to minimize the damage caused by COVID-19, established infection control and public health measures need to be followed strictly. Particularly, the WHO guidelines issued during the spread of SARS-COV and MERS-COV regarding the control of infections greatly reduced the risk of transmission of acute respiratory infections, including SARS-CoV-2. These guidelines involve avoiding close contact with people suffering from acute respiratory infections, frequent hand-washing, especially after direct contact with ill people or their environment, and avoiding unprotected contact with farm or wild animals [143]. In addition, people with symptoms of COVID-19 should follow standard cough etiquette such as maintaining distance, covering of mouth with hand or disposable tissue or clothing while coughing or sneezing, regular washing of hands, etc. [13]. While social distancing and good hand hygiene are the most important methods to prevent virus transmission, some countries have also issued new guidelines of mandatory mask wearing by healthy individuals in public settings, particularly in places where physical distancing is difficult [144]. Governments and regulatory authorities are, however, facing a number of difficulties in implementing the guidelines to contain the virus. 

**(A) Masks, to Use or Not to Use:** Various studies have indicated that the spread of coronavirus is also reported rampantly by asymptomatic carriers [145]. In these circumstances, wearing a face mask potentially minimizes the spread of infection to healthy individuals by infected persons who may not know that they have infection (asymptomatic carriers). After the outbreak of COVID-19, wearing a face mask was widely practiced in China and other Asian countries such as South Korea, Taiwan, Japan, etc. Large differences in opinions were observed on the use of masks by the general public and community settings, even though authorities across the globe agreed to recommend the use of masks for infected individuals and healthcare professionals [146]. Therefore, different guidelines were issued by the governments of different countries. For instance, initially, the WHO recommended use of mask by only those who are taking care of infected individuals but later advised the use even for healthy individuals in community settings [147]. In the USA, Centers for Disease Control and Prevention does not recommend a face mask. However, later on, several states including Washington, D.C. have issued mandates for the use of a face mask in public [148]. The United Kingdom strictly recommended using a face mask in hospitals but is not convinced about the widespread benefits of wearing a mask for the general public due to the lack of sufficient evidence [149]. On the other hand, authorities in Germany have suggested that recommendation of face masks for the general public may create a false sense of security and might lead to neglecting fundamental hygiene measures, such as proper hand hygiene [150]. Notably, various pieces of evidence suggested that the transmission of SARS-CoV-2 is possible even before the onset of symptoms; therefore, recommendation of face masks for everyone including infected individuals can be very helpful in reducing the potential community transmission [151,152]. 

**(B) Difficulties in Convincing People:** Since no specific therapeutics or vaccines are available, this leaves prophylaxis as the only measure to control the spread of the disease. These measures include self-isolation, quarantine, social distancing, and community containment. Particularly, self-isolation and social distancing are effective methods to slow the transmission of SARS-CoV-2. However, in various countries including USA, Australia, India, Brazil, and many other European countries, most people are ignoring public health advisories and guidelines released by governments and various health organizations across the world, including the WHO, the UK’s NHS, the US’ CDC (Centers for Disease Control and Prevention), etc. In some places, not only have younger individuals refused to follow social distancing norms but even people aged above 60 seemed less concerned about the risks. Furthermore, even among the people who understand the importance of public health measures very well, such as washing hands, maintaining appropriate distance, avoiding touching face, etc., people fail to practice them efficiently [153]. One of the reasons for the carelessness is the lack of past experiences to understand such a crisis. To convenience the people who are complacent about COVID-19, health psychologists suggest to adopt creative ways of picturing the world months or years after the pandemic [154]. Since some of the symptoms of common cold and COVID-19 are similar, it remains difficult for many to understand the possible threats of COVID-19. Authorities and health personnel must strive hard to convince the population that the threat of coronavirus is very much real and not hypothetical and that COVID-19 is different from the common cold and can be fatal. 

**(c) Cultural and Socio-economic Stigmas:** According to the WHO, social stigma in a health crisis relates to the negative association between a person or group of people who share certain characteristics and a specific disease. For instance, during a pandemic, infected people can be stereotyped, negatively labeled, discriminated against, and/or treated differently due to a perceived link with the disease [155]. Such type of discrimination can have negative psychological effects on the infected individuals as well as their caregivers, family, friends, and communities. In addition, people who are not infected but are associated with these people may also suffer from social stigma. The current COVID-19 pandemic has promoted social stigma and discrimination against people of certain ethnic backgrounds as well as anyone perceived to have been in contact with the virus. Particularly, many of the facts about COVID-19 are still unknown; therefore, people are very scared, and this creates confusion, anxiety, and fear among the general public, which possibly fuels dangerous stereotypes [156]. 

Stigma associated with COVID-19 can disturb the social fabric of society and also promotes self-isolation of affected groups, which can accelerate the spread of virus and create serious health problems since stigma may encourage infected individuals to hide their disease due to the fear of discrimination, inhibit people from seeking medical care, and discourage them from following public health guidelines [157]. Studies suggest that fear and stigma during the spread of infectious diseases can seriously impede the response of the authorities [158]. Therefore, various positive steps such as providing proper healthcare services, building trust, and showing empathy with infected individuals are highly required to encourage people to seek medical care and keep themselves safe [159]. Moreover, the general public should be educated to avoid stereotyping infected individuals, creating false narratives about the disease, and dehumanizing those who are infected and to avoid naming the disease which associates a geographical location, an individual, or a group of people. Both print and electronic media can significantly influence the perception of the people regarding the infected individuals and affected communities. Therefore, words must be carefully chosen while communicating news about the disease, and those words which may create stigma against specific group of people must be avoided. Particularly, quarantined people are most likely to face stigmatization and social rejection; therefore, efforts are required for properly educating people about the disease and rationale for quarantine [160]. 

## 11. Were We Caught Unprepared for This Scale of Crisis?

So far, a variety of viruses have shown pandemic potential, including influenza and other coronaviruses such as SARS, MERS, and SARS-CoV-2. However, the persistence, versatility, potential severity, and speed of transmission of the Spanish flu (influenza), one of the deadliest pandemics of the 20th century, can only be matched with the current COVID-19 pandemic [161]. For several decades, random outbreaks of severe influenza and coronaviruses have occurred which have caused several deaths. These viruses, such as influenza A viruses, including H5N1, H7N9, H10N8. etc., and coronaviruses, such as SARS-CoV-1 and MERS-CoV, all had prospective ability to cause huge pandemics. Although, the likelihood of an outbreak causing a potential pandemic is often difficult to judge due to limited surveillance and uncertain behavior of the virus. However, the data collected from past pandemics of influenza and coronaviruses may have provided various clues for planning for future pandemics. 

Particularly, with the recent advancement of biologic and epidemiologic knowledge, future pandemics can be predicted at least with some degree of certainty. With the advent of artificial intelligence, web-based generated field reports and analysis of search patterns can potentially provide valuable intelligence which can offer important clues about the next emerging pandemic [162,163]. Indeed, various intellectuals have been warning about the unpreparedness of the global community in dealing with viral pandemics. For example, Bill Gates, in one his famous TED talks in 2015, said the following, “If anything kills over 10 million people in the next few decades, it’s most likely to be a highly infectious virus, rather than a war. Not missiles, but microbes. Now, part of this reason is that we’ve invested a huge amount in nuclear deterrents, but we’ve actually invested very little in a system to stop an epidemic. We’re not ready for the next epidemic.” (Bill Gates, TEDx, Vancouver, British Columbia, Canada, 2015). 

After the 1997 influenza outbreak in Hong Kong, the WHO had formulated formal guidelines for dealing with situations during a global pandemic. These guidelines specifically include two strategic steps: (i) risk assessment, which involves two components—data collection (investigating the circumstances of the initial infection and subsequent infections and searching for further evidence of spread) and data evaluation (interpreting and communicating the significance of the threat based on the available data); and (ii) risk management, which is a process of continuously considering and updating alternative courses of action as new action is obtained, defining potential risks and benefits of each approach, and selecting the next step, or series of steps, recommended for appropriate authorities [164]. Only very few countries committed staff for collecting the information needed for risk assessment of future pandemics. Thereafter, several other recommendations have been issued by the WHO to warn about future outbreaks of viral pandemics, but most of the threats were largely ignored by the global community [165].

## 12. Were Earlier Warnings Ignored?

In the cases of coronaviruses, like SARS-CoV-2, the other two recently emerged pandemics related to SARS and MERS were zoonoses, and critical understanding of these two pandemics could have been crucial to plan countermeasures toward identifying, managing, and preventing future outbreaks. Still, there has been no significant and sustained progress in the development of countermeasures in dealing with pandemics, including antivirals and vaccines to treat and prevent coronavirus infection, respectively [166]. Although there have been seven outbreaks of acute respiratory disease associated with coronaviruses since 1960, not much has been learned from these instances. After the SARS outbreak, there has been very slow progress towards the development of both vaccines and antiviral medicines. Notable successes have been achieved in developing broad-spectrum antivirals against Ebola and HIV virus. Indeed, these antivirals are now being currently tested both in vitro and in animal models for efficacy against COVID-19. Although vaccine development methods have been tested, including whole inactivated virus, live attenuated virus, mRNA and DNA approaches, recombinant viral protein, and nanoparticle approaches, for last eighteen years, none of these products have passed past phase I studies. Indeed, one of the possible reasons for the slow progress is the scarcity of research funding, which has been hardly noticed by the general public, media fraternity, and government authorities across the globe until the outbreak of the COVID-19 pandemic. All types of coronaviruses should be of great concern to the global community; therefore, sincere efforts by healthcare professionals and heavy investments by government authorities are required to prepare effective countermeasures for dealing with such future pandemics. 

## 13. Lessons for the Future: Proposed Strategies

For last two decades, three major pandemics associated with coronaviruses occurred randomly at irregular intervals. Still, it has become clear by now that medical, political, and scientific communities across the globe are not sufficiently prepared to deal with another outbreak of a pathogenic virus. The scientific knowledge gained by handling the first coronavirus pandemic (SARS) and the subsequent advancement of medical science helped us significantly to successfully deal with the next pandemic (MERS), which was largely contained before it caused major mortalities. Despite this, the global community was caught largely unprepared when the COVID-19 pandemic occurred at the end of 2019. This clearly demonstrates that the scientific knowledge about coronaviruses which we have gained so far appears to be much, but still there are many things to be learned. Human pandemics largely emerge due to various genetic mechanisms with varied severity. Particularly, pandemics associated with human coronavirus have emerged due to the evolution of viruses which have caused earlier outbreaks through the transmission between viral reservoirs within other animal hosts. Once again, the COVID-19 outbreak has taught us many lessons about zoonotic reservoirs; therefore, further understanding of animal models may provide vital information for unravelling the viral pathogenicity and rational designing of therapeutics for any future pathogenic viral outbreaks. So far, the data collected on genetic evolution, receptor binding, and pathogenesis for all three coronavirus, including SARS-COV-2, have led to the recognition of bats as the common natural origin. 

Although most of the symptoms of the disease caused by all three coronaviruses appeared to be same, the rapid spread of COVID-19 disease makes it more lethal than other pandemics. In addition, the adaptation of the S glycoprotein and its affinity for ACE2 has significantly enhanced the severity of COVID-19 infection. Therefore, a prospective vaccine containing S glycoproteins and inactivated SARS-CoV-2 may prove to be helpful in preventing infection. Furthermore, intensive research on the genetic diversity and recombination events of SARS-CoV-2 is highly desirable to produce an effective vaccine or therapeutics. In the absence of complete structural and life cycle details of the virus, and considering the lethal nature of the current SARS-CoV-2 infection, there is an immense demand for the development of suitable therapeutics. So far, existing medicines have only been effective in partial handling of the symptoms and no definite lines of treatment and medicines have been defined so far. 

Currently, the ideas for the development of medicines are mainly dependent on the mechanism of the viral infection, i.e., virus entry into the cells and its multiplication using the cellular machinery of the host cells along with the damage of the host cells. Among the various drugs which have been tested for controlling COVID-19, previously used antiviral drugs for similar kind of infections were also included. Other prospective medicine under consideration or being developed includes entry inhibitors, replication inhibitors, protease inhibitor, antibody- or antigen fragments-based therapeutics, herbal medicines, and so on. However, only a few of them have been found to produce promising results for the treatment of COVID-19 such as hydroxychloroquine, dexamethasone, remdesivir, lopinavir, and ritonavir. Besides, very recently, some vaccines have been developed and approved for prophylaxis including the Pfizer-BioNTech COVID-19 vaccine, Moderna’s COVID-19 vaccine, Sinovac, and Sputnik V. Nevertheless, a complete effective treatment of the infection is still unavailable; however, potential strategies are emerging rapidly. Clearly, under these circumstances, highly aggressive measures are required to stop the transmission of virus and to minimize the damage. The prevention strategies are mostly restricted to two options, including strict social distancing measures, such as travel ban, closure of public places, and restricted economic activities, and the other option is to wait for the uncertain development of ‘herd immunity’ or vaccine-derived immunity [167]. Most of the countries across the globe have adopted the former strategy of strict social distancing guidelines, since the prevention of viral transmission is the key to reducing the burden of COVID-19. 

This strategy includes various measures: one of the most important steps is the preservation and strengthening of healthcare systems. This includes strict execution of infection control measures in hospitals to protect healthcare workers, maintaining adequate strength of work force for the prevention of hospital outbreaks which often occur during such community pandemics. Maintaining adequate supplies of personal protective equipment (PPE) for hospital staff can significantly decrease the number of nosocomial transmissions of disease. Patient management should be monitored effectively to provide effective treatment and/or to avoid overtreatment of infected individuals. Steps must be taken to encourage people to follow public health measures, i.e., proper following of isolation or quarantine advice and maintaining social distance, etc. 

As COVID-19 has already spread across the world, the process of finding a solution must involve the efforts of entire global community; therefore, a lack of trust among countries may adversely affect scientific collaboration and jeopardize the research process for controlling the disease. Panic-control measures must be taken to avoid spreading social disruptions which often occur under these circumstances. Excessive control measures must be avoided which can potentially enhance public frustration, invoke false feeling of safety, and destabilize the economy. Investigation is still ongoing to determine the real cause of SARS-CoV-2 transmission; however, it is largely believed that the virus is transmitted to humans via infected live animals, particularly bats. Therefore, a complete ban must be enforced on the consumption of wild animals as a source of food. Indeed, after experiencing the devastating effect of three major coronaviruses pandemics, the global community may now make extra efforts for preventing the transmission of animal viruses to humans and remain vigilant, should it happen again in the future. Although future evolution of such pandemic remains unpredictable, strict adherence to the classic public health guidelines is highly imperative. Nevertheless, the COVID-19 outbreak in this century once again underscores the enduring threat of infectious diseases spread by pathogenic viruses to humanity, which require effective global co-operation and a high level of preparedness. 

## Figures and Tables

**Table 1:** Top 10 country-wise analysis of the count of COVID-19 related cases.

S.No.	Country	Total Cases	Total Deaths	Total Recovered	Active Cases *	Serious Critical *
1	USA	9,802,374	239,842	6,293,132	3,269,400	18,045
2	India	8,364,086	124,354	7,711,809	527,923	8944
3	Brazil	5,590,941	161,170	5,064,344	365,427	8318
4	Russia	1,712,858	29,509	1,279,169	404,180	2300
5	France	1,543,321	38,674	122,662	1,381,985	4089
6	Spain	1,356,798	38,118	N/A	N/A	2786
7	Argentina	1,205,928	32,520	1,017,647	155,761	4816
8	Colombia	1,108,084	32,013	1,002,202	73,869	2376
9	UK	1,099,059	47,742	N/A	N/A	1142
10	Mexico	943,630	93,228	697,402	153,000	2838

* As of 5 November 2020.
